# Early Visual Processing Is Associated With Social Cognitive Performance in Recent-Onset Schizophrenia

**DOI:** 10.3389/fpsyt.2020.00823

**Published:** 2020-08-25

**Authors:** Amanda McCleery, Jonathan K. Wynn, Junghee Lee, Eric A. Reavis, Joseph Ventura, Kenneth L. Subotnik, Michael F. Green, Keith H. Nuechterlein

**Affiliations:** ^1^Department of Psychological and Brain Sciences, University of Iowa, Iowa City, IA, United States; ^2^Semel Institute for Neuroscience and Human Behavior, University of California–Los Angeles, Los Angeles, CA, United States; ^3^Mental Illness Research, Education and Clinical Center (MIRECC), VA Greater Los Angeles Healthcare System, Los Angeles, CA, United States; ^4^Department of Psychiatry and Behavioral Neurology, University of Alabama at Birmingham, Birmingham, AL, United States; ^5^Department of Psychology, University of California–Los Angeles, Los Angeles, CA, United States

**Keywords:** visual perception, backward masking, theory of mind, emotion identification, first episode psychosis

## Abstract

**Background:**

Early-stage visual processing deficits are evident in chronic schizophrenia. Consistent with a cascade model of information processing, whereby early perceptual processes have downstream effects on higher-order cognition, impaired visual processing is associated with deficits in social cognition in this clinical population. However, the nature of this relationship in the early phase of illness is unknown. Here, we present data from a study of early visual processing and social cognitive performance in recent-onset schizophrenia (ROSz).

**Method:**

Thirty-two people with ROSz and 20 healthy controls (HC) completed a visual backward masking task using stimuli of real world objects (Object Masking) to assess early-stage (i.e., 0–125 ms post-stimulus onset) visual processing. Subjects also completed two tasks of social cognition, one assessing relatively low-level processes of emotion identification (Emotion Biological Motion, EmoBio), and another assessing more complex, higher-order theory of mind abilities (The Awareness of Social Inference Test, TASIT). Group differences were tested with repeated measures ANOVAs and *t*-tests. Bivariate correlations and linear regressions tested the strength of associations between early-stage visual processing and social cognitive performance in ROSz.

**Results:**

For Object Masking, the mask interfered with object identification over a longer interval for ROSz than for HC [*F* (3.19, 159.35) = 8.51, *p <* 0.001]. ROSz were less accurate on the EmoBio task [*t* (50) = −3.36, *p* = 0.001] and on the TASIT compared to HC [*F* (1, 50) = 38.37, *p* < 0.001]. For the TASIT ROSz were disproportionately impaired on items assessing sarcasm detection [*F* (1, 50) = 4.30, *p* = 0.04]. In ROSz, better Object Masking performance was associated with better social cognitive performance [*r*_EmoBio_ = 0.45, *p* < 0.01; *r*_TASIT_ = 0.41, *p* < 0.02]. Regression analyses did not provide significant support for low-level social cognition mediating the relationship between visual processing and high-level social cognition.

**Conclusion:**

Early-stage visual processing, low-level social cognition, and high-level social cognition were all significantly impaired in ROSz. Early-stage visual processing was associated with performance on the social cognitive tasks in ROSz, consistent with a cascade model of information processing. However, significant cascading effects within social cognition were not supported. These data suggest that interventions directed at early visual processing may yield downstream effects on social cognitive processes.

## Introduction

Schizophrenia is associated with marked impairment across a variety of information processing domains, spanning from very early stages of perceptual processing, through complex, higher-order cognitive processes. Deficits in early-stage visual processing have consistently been reported in chronic schizophrenia (1–6). Consistent with a cascade model of information processing, whereby disruptions in early perceptual processes have downstream consequences for higher-order cognition and functioning, impaired visual processing is associated with deficits in social cognition (i.e., processing of social stimuli including emotion identification and mental state attribution) in this clinical population (7–14). Moreover, structural equation modeling analyses have demonstrated that social cognition mediates the relationship between visual processing and community functioning in people with schizophrenia (7, 12, 15). Thus, visual processing and social cognitive abilities are important components of the pathway toward functional recovery in schizophrenia.

Social cognitive deficits are well-documented in people with recent-onset schizophrenia (ROSz), with meta-analytic reviews reporting large effect sizes that are on par with those obtained from chronic phase schizophrenia samples (16–18). In contrast, considerably less is known about visual perception abnormalities in the early phase of illness. Similarly, the nature of the relationship between visual processing and social cognition in the early phase of illness is unknown. The available evidence strongly suggests visual processing abnormalities in ROSz (19–23), with patients exhibiting significantly impaired contour integration (19), visual perception organization (20), and motion processing (21) relative to healthy adults. However, the magnitude of impairment may be attenuated relative to what is observed in chronic phase schizophrenia (19, 24, 25).

The earliest stages of visual processing can be probed behaviorally with visual masking tasks (see (26) for a comprehensive review). In these tasks, a rapidly presented target stimulus is either shortly preceded by (for forward masking) or shortly followed by (for backward masking) a masking stimulus which interferes with processing of the target. Depending on the type of paradigm used, the masking stimuli may spatially overlap the target, or it may surround, but not touch, the target. The duration of the interval between the target and mask is brief (i.e., 0–500 ms), and is varied across trials. Accuracy for identifying the target, or some aspect of the target (e.g., target location, a feature of the target), is assessed yielding a masking function. For backward masking, the typical response function is S-shaped, with very poor accuracy at short intervals between target and mask, and improved performance as the interval between target and mask increases.

Prior studies of visual backward masking in ROSz indicate impaired performance relative to healthy adults (22, 25), an association with duration of untreated psychosis (i.e., short duration of untreated psychosis associated with better performance; (27)), and stability of performance over 6–24 months (22, 23). Here, we assessed visual backward masking and test the association between early visual processing and performance on social cognitive tasks that involve processing of visual cues in people with ROSz and healthy adults. We hypothesized that visual backward masking performance and social cognitive task performance would be significantly impaired in the ROSz sample compared to healthy adults. In addition, we hypothesized that visual backward masking performance would be significantly correlated with social cognitive task performance in the patient group.

## Method

### Participants

Thirty-two people with recent-onset schizophrenia (ROSz) and 20 healthy controls (HC) participated in this study. The patient participants were recruited from the UCLA Aftercare Research Program, an outpatient research clinic for ROSz. Inclusion criteria were: 1) onset of a first psychotic episode within 24 months of program entry, 2) fulfillment of DSM-IV (28) criteria for schizophrenia, schizoaffective disorder, depressed type, or schizophreniform disorder, 3) age of 18 to 45 years, and 4) sufficient fluency in English to allow for valid completion of the testing protocol. These participants met criteria for schizophrenia (*n* = 24), schizoaffective disorder, depressed type (*n* = 3), or schizophreniform disorder (*n* = 5). All ROSz participants were prescribed atypical antipsychotic medication, and chlorpromazine (CPZ) equivalent dosing information (29, 30) can be found in **Table 1**.

**Table 1:** Demographic characteristics of the study participants.

	ROSz (*n* = 32)	HC (*n* = 20)	
	n (%)	n (%)	χ^2^ (*df*), *p*-value
Gender (male)	22 (69%)	15 (75%)	χ^2^ (1) = 0.23, *p* = 0.63, Cramer’s *V* = 0.02
Race			
Caucasian	10 (31%)	6 (30%)	χ^2^ (4) = 1.99, *p* = 0.74, Cramer’s *V* = 0.20
African American	8 (25%)	4 (20%)	
Asian	2 (6%)	2 (10%)	
Native American	0 (0%)	1 (5%)	
Other	12 (38%)	7 (35%)	
Ethnicity (Hispanic)	14 (44%)	10 (50%)	χ^2^ (1) = 0.02, *p* = 0.89, Cramer’s *V* = 0.06
	mean (*s.d.*)	mean (*s.d.*)	*t*(*df*), *p*-value
Age	23.71 (4.07)	23.05 (2.89)	*t*(50) = 0.63, *p* = 0.53, Cohen’s *d* = 0.18
Personal education	12.29 (1.40)	14.50 (1.36)	*t*(49) = −5.58, *p* < 0.001, Cohen’s *d* = −1.59
Parental education	14.34 (3.97)	14.15 (3.07)	*t*(47) = 0.19, *p* = 0.85, Cohen’s *d* = 0.06
MCCB neurocognitive composite score	28.73 (15.76)	48.16 (6.60)	*t*(50) = 6.20, *p* < 0.001, Cohen’s *d* = 1.75
BPRS positive symptoms	13.28 (6.13)		
BPRS negative symptoms	6.56 (3.38)		
BPRS total score	37.13 (9.94)		
Antipsychotic medication dosing (CPZ equivalents)	304.94 (143.82)		

The HC sample were recruited *via* advertisements (e.g., online classified ads, flyers in the community), and had no current psychiatric diagnosis, no lifetime history of any psychotic disorder, bipolar disorder, or recurrent depressive disorder, and no history of a psychotic disorder among their first degree relatives. For all subjects, current substance or alcohol use disorder, history of head injury with loss of consciousness, seizure disorder, and/or IQ below 70 were exclusionary. Demographic information for the study participants is presented in **Table 1**.

### Clinical and Cognitive Characterization

DSM-IV diagnoses were made using the Structured Clinical Interview for DSM-IV (SCID) (31) and SCID-II (32). For the patient sample, current (i.e., within the 2 week period prior to testing) psychiatric symptoms were assessed by trained raters with the 24-item Brief Psychiatric Rating Scale (BPRS) (33). Each clinical rater achieved a median Intraclass Correlation Coefficient (ICC) of 0.80 or higher across all BPRS items compared with the criterion ratings and participated in a quality assurance program (34). For the SCID, clinical raters demonstrated an overall kappa coefficient, kappa sensitivity, and kappa specificity of 0.75 or greater, and a diagnostic accuracy kappa coefficient of 0.85 or greater. For all participants, cognitive performance was assessed using the MATRICS Consensus Cognitive Battery (MCCB) (35). The variable of interest was the age and gender-corrected neurocognitive composite score, which reflects performance on tasks assessing speed of processing, attention/vigilance, working memory, verbal learning, visual learning, and reasoning and problem solving.

### Visual Processing

**Figure 1:**
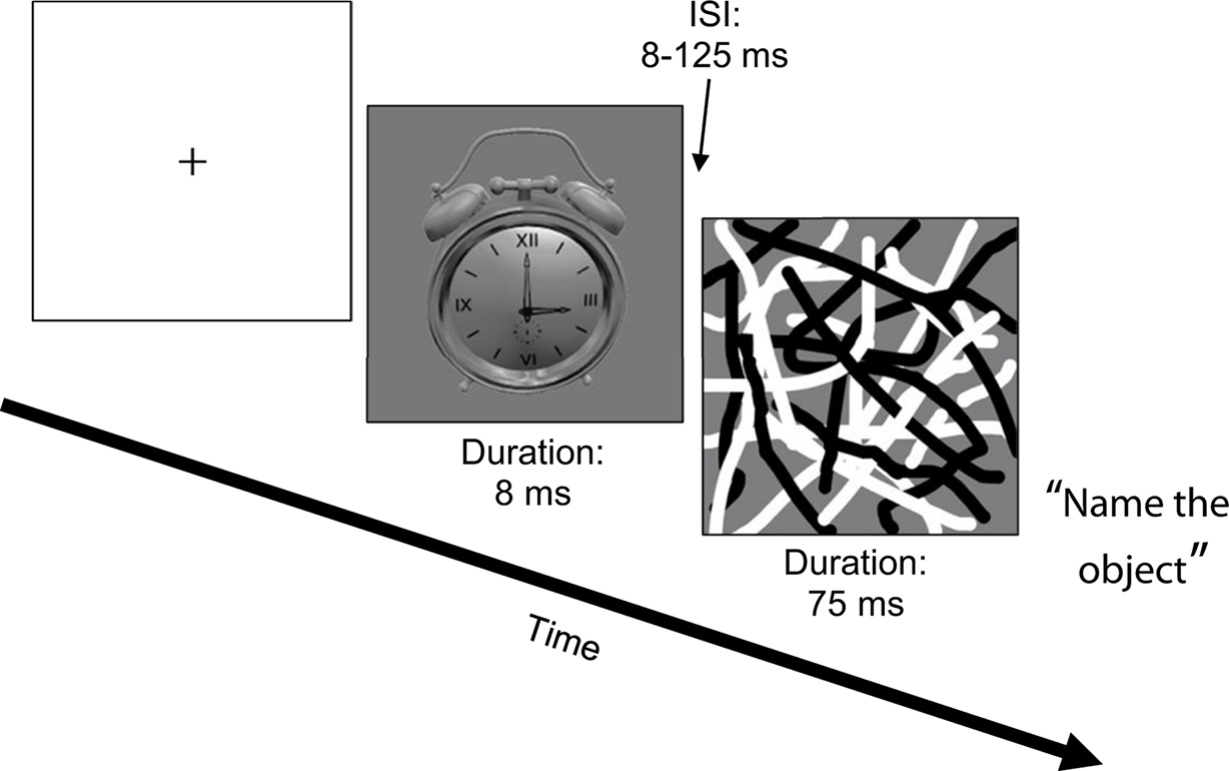
Object Masking task.

All participants completed a backward masking task using common household objects as targets (“Object Masking”; see **Figure 1**) (36). The task was run in Eprime (Psychology Software Tools, Inc., Sharpsburg, PA, USA) on a Dell Alienware Aurora R4 Intel Core i7 PC, using a Nvidia GeForce GTX 560 Ti video card. All stimuli were presented on a 120 Hz Asus V0236 LCD 23” monitor. Target stimuli from one of six different objects were presented for 8 ms. Target stimuli were followed by a masking stimulus (overlapping black and white curved lines that spatially overlapped the target location) that was presented for 75 ms after a variable inter-stimulus interval (ISI) of 8–125 ms (in 17 ms increments). There were 12 trials per ISI, plus 12 unmasked trials (i.e., target was presented without a mask). After each trial, a list of the six objects appeared and participants verbally reported which object they thought the target was and the tester entered the response into the computer. Because participants gave verbal responses, reaction times were not recorded. The dependent measure was accuracy, measured as proportion correct. Mean accuracy across ISIs was the variable of interest for the correlation and regression analyses (10).

### Social Cognition

Low-level emotion identification was assessed with Emotion in Biological Motion (EmoBio) (37), and higher-order theory of mind abilities were assessed with Part 3 of The Awareness of Social Inference Test (TASIT) (38). For EmoBio, the ability to perceive emotion based on limited cues from body motion (i.e., gait, limb movement, speed of movement) was assessed using the point-light walker stimuli (39) and adapted for clinical trials use (37). Twenty-four point-light walker clips of 5–10 s in length were presented on a computer screen. Participants were asked which of five emotional states (fear, anger, happiness, sadness, or neutral) best described the movement of the walker. The five choices for emotional state were presented on the computer screen immediately after presentation of the clip. The dependent measure was accuracy, measured as proportion correct.

The TASIT Part 3 is comprised of 16 audio-video vignettes of adults interacting with each other. After each vignette, participants respond to four yes/no questions probing the beliefs and intentions of the characters. Half of the vignettes involve a character lying to another character, and half of the vignettes involve a character making sarcastic comments. Participants detect lies and sarcasm based on integration of multiple sources of social information including facial affect, vocal prosody, body motion, mental state attributions, social knowledge, and contextual cues. The dependent measure was accuracy, measured as proportion correct for the Lies and Sarcasm conditions.

### Data Analysis

The data were screened for univariate outliers using box plots, and for skewness with histograms. Group differences for the visual processing and social cognition tasks were tested with repeated measures ANOVAs and *t*-tests. When assumptions of sphericity were violated in the repeated measures analyses, a Greenhouse-Geisser correction was used. Bonferroni corrections were used to control the familywise error rate of the *post-hoc* analyses. For the *post-hoc* paired *t*-tests of the Object Masking data, critical α = 0.05/14 tests or 0.004. For *post-hoc t*-tests of the TASIT data, critical α = 0.05/4 tests or 0.01. In the ROSz group, bivariate correlations and multiple linear regression (method = enter) tested the strength of associations between early-stage visual processing and social cognitive task performance. For the regression analyses, the data were screened for multivariate outliers using Mahalanobis distances (critical α = 0.001). Effect sizes were interpreted as small (*r* = 0.10, partial η^2^ = 0.01, Cohen’s *d* = 0.20), medium (*r* = 0.30, partial η^2^ = 0.09, Cohen’s *d* = 0.50), or large (*r* = 0.50, partial η^2^ = 0.25, Cohen’s *d* = 0.80) (40, 41).

## Results

### Sample Characteristics

Demographic, clinical, and cognitive characteristics of the study participants are presented in **Table 1**. The two groups were well-matched on demographic characteristics, including age, gender, race and ethnicity, and level of parental education. Twenty-two (69%) of the ROSz participants met criteria for positive symptom remission (i.e., score of ≤3 on BPRS hallucinations, unusual thought content, and conceptual disorganization) for the 2 week period prior to testing (42). As expected, the ROSz participants exhibited significant neurocognitive impairment relative to the HC group [*t* (50) = 6.20, *p* < 0.001, Cohen’s *d* = 1.75]. The magnitude of impairment was similar to our previous findings with a different ROSz patient cohort (43).

### Visual Processing

**Figure 2:**
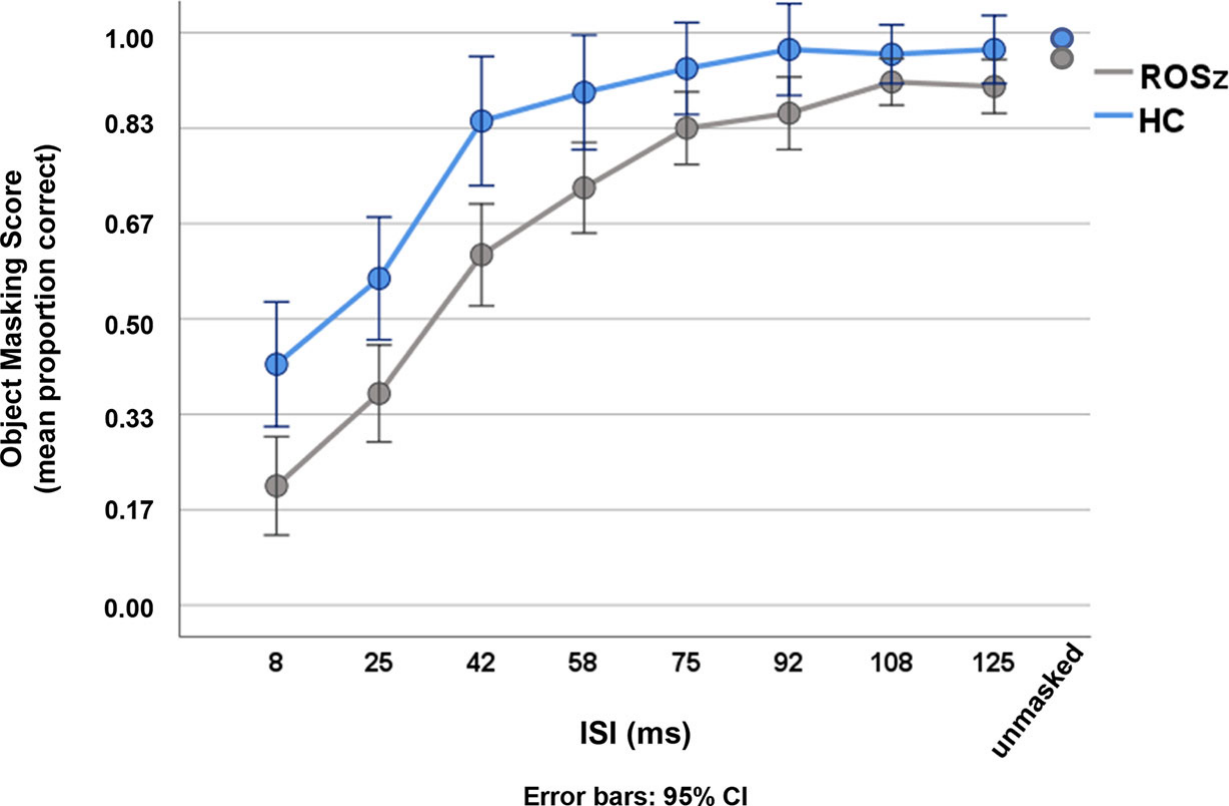
Object Masking performance in recent-onset schizophrenia and healthy controls.

Results for the Object Masking task are presented in **Figure 2** and **Table 2**. Mean accuracy (i.e., proportion correct) for the unmasked control condition exceeded 0.90, and performance did not significantly differ between groups [*p* = 0.12, Cohen’s *d* = 0.47]. Data for the individual ISIs were negatively skewed, as is typical for backward masking tasks, and were thus log transformed prior to repeated measures analysis. Across ISIs, a typical backward masking response profile was evident [main effect of ISI: *F* (3.19, 159.35) = 44.08, *p* < 0.001, partial η^2^ = 0.47]. Accuracy was poor at brief ISIs [8 ms ISI mean accuracy = 0.32, *s.d.* = 0.25], indicating that the mask interfered with processing of the target stimulus, and performance steadily improved as the ISI duration increased [125 ms ISI mean accuracy = 0.94, *s.d.* = 0.14], reflecting escape from the masking effect.

**Table 2:** Descriptive statistics for visual processing and social cognitive task performance.

	ROSz mean (*s.d.*)	HC mean (*s.d.*)	Contrast *F* or *t* (*df*), *p*-value
Object Masking ISI			
Unmasked	0.95 (0.09)	0.98 (0.06)	Group: *t* (50) = −1.58, *p* = 0.12, Cohen’s *d* = 0.47
8 ms	0.21 (0.21)	0.42 (0.29)	
25 ms	0.37 (0.21)	0.57 (0.28)	
42 ms	0.61 (0.28)	0.85 (0.19)	
58 ms	0.72 (0.26)	0.90 (0.15)	
75 ms	0.83 (0.22)	0.94 (0.09)	
92 ms	0.86 (0.22)	0.97 (0.08)	
108 ms	0.91 (0.14)	0.96 (0.06)	
125 ms	0.91 (0.16)	0.97 (0.05)	
			Main effect of ISI: *F* (3.19, 159.35) = 44.08, *p* < 0.001, partial η^2^ = 0.47
			Main effect of group: *F* (1,50) = 5.76, *p* = 0.02, partial η^2^ = 0.10
			ISI x group: *F* (3.19, 159.35) = 8.51, *p* < 0.001, partial η^2^ = 0.15
EmoBio	0.78 (0.08)	0.86 (0.05)	Group: *t* (50) = −3.66, p = p=0.001, Cohen’s *d* = 1.10
TASIT Lies	0.79 (0.10)	0.88 (0.07)	
TASIT Sarcasm	0.70 (0.14)	0.88 (0.08)	
			Main effect of condition: *F* (1, 50) = 4.94, *p* = 0.03, partial η^2^ = 0.09
			Main effect of group: *F* (1, 50) = 38.37, *p* < 0.001, partial η^2^ = 0.43
			Condition x group: *F* (1, 50) = 4.30, *p* = 0.04, partial η^2^ = 0.08

The interference by the mask was greater in ROSz compared to HC [group × ISI: *F* (3.19, 159.35) = 8.51, *p* < 0.001, partial η^2^ = 0.15]. For the HC group, follow-up paired *t*-tests indicated that accuracy significantly improved between 25 and 42 ms ISI [*t* (19) = 6.52, *p* < 0.001, Cohen’s *d* = 1.45], but tapered off between 42 and 58 ms ISI [*t* (19) = 1.87, *p* = 0.07, Cohen’s *d* = 0.19], reflecting escape from the masking effect. Compared to HC, the ROSz group exhibited worse performance across ISIs [group: *F* (1, 50) = 5.76, *p* = 0.02, partial η^2^ = 0.10]. Follow-up paired *t*-tests indicated that performance in the patient group steadily improved through 75 ms ISI [*p*’s < 0.001, Cohen’s *d*’s ≥ 0.75], before leveling off between 75 and 92 ms ISI [*t* (31) = 1.57, *p* = 0.13, Cohen’s d = 0.28] reflecting escape from the masking effect.^1^

### Social Cognition

**Figure 3:**
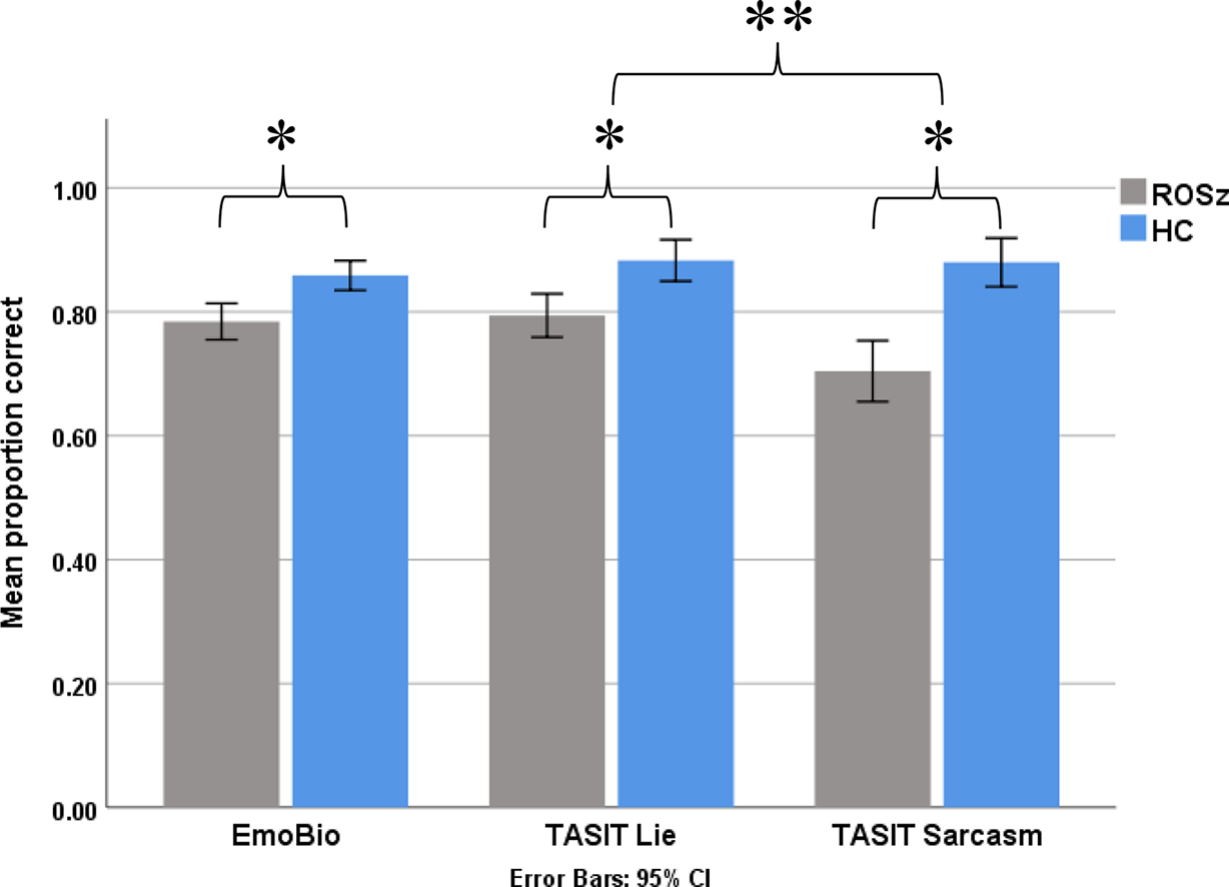
Social cognitive task performance in recent-onset schizophrenia and healthy controls. *Denotes group contrast p < 0.05. **Denotes group × condition interaction effect p < 0.05.

Results for the social cognitive tasks are presented in **Figure 3** and **Table 2**. Scores were normally distributed and free of outliers. For the EmoBio task, the ROSz group were less accurate compared to HC [*t* (50) = 3.36, *p* = 0.001, Cohen’s *d* = 1.10]. Similarly, for the TASIT, the ROSz group exhibited overall poorer performance compared to HC [*F* (1,50) = 38.37, *p* < 0.001, partial η^2^ = 0.43]. There was a significant interaction between TASIT condition and group [*F* (1,50) = 4.30, *p* = 0.04, partial η^2^ = 0.08]. Compared to HC, ROSz exhibited impaired performance on lie [*t* (50) = −3.54, *p* = 0.001, Cohen’s *d* = −1.00] and sarcasm [*t* (50) = −5.17, *p* < 0.001, Cohen’s *d* = −1.46] detection. However, the ROSz group were disproportionately impaired on items assessing sarcasm detection [*t* (30) = 3.09, *p* = 0.004, Cohen’s *d* = 0.55] compared to HC [*t* (19) = 0.13, *p* = 0.90, Cohen’s *d* = 0.03].

### Relationship Between Visual Processing and Social Cognition

**Figure 4:**
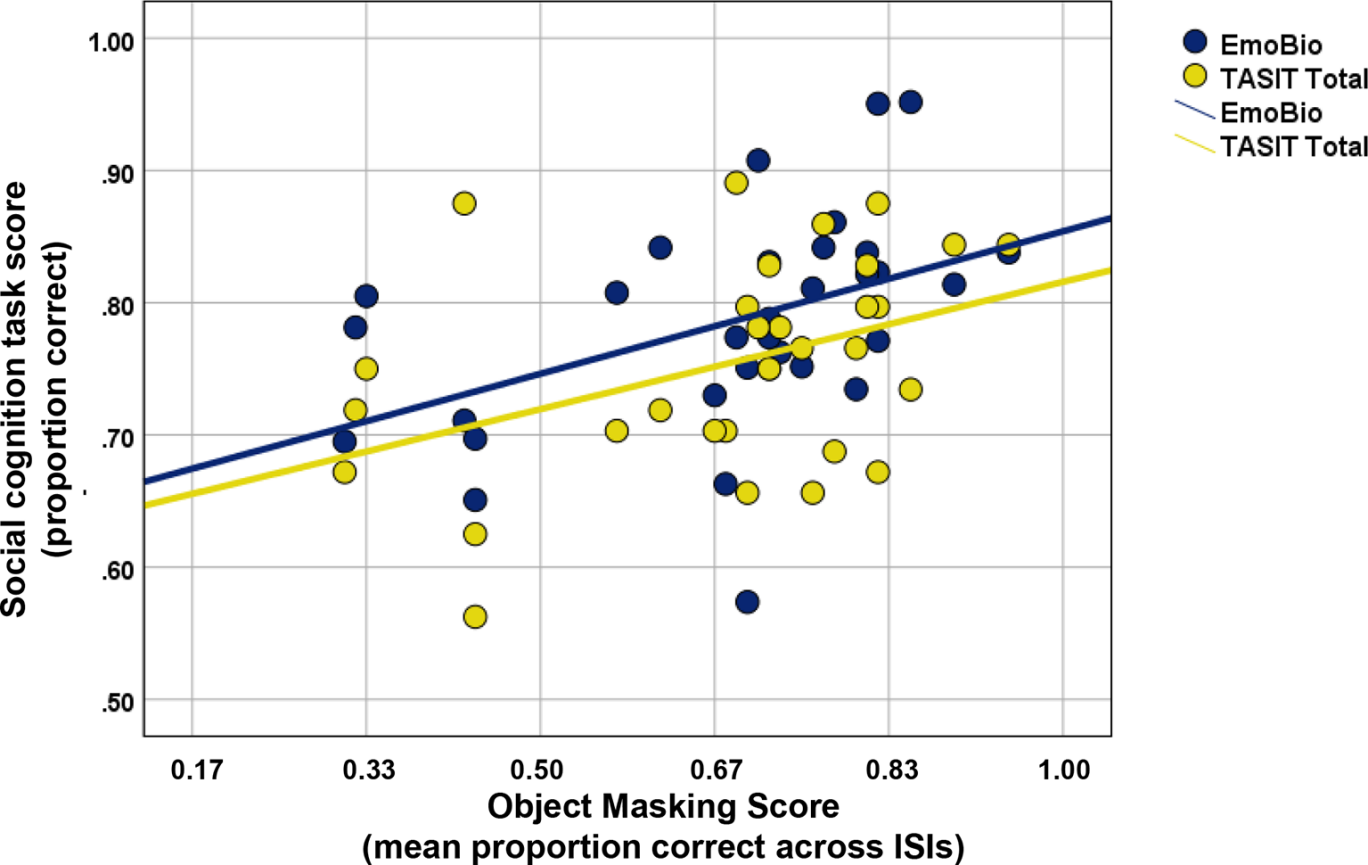
Scatterplot of Object Masking performance and social cognitive task performance in recent-onset schizophrenia.

A scatterplot of the relationship between Object Masking performance and social cognitive task performance in the patient sample is displayed in **Figure 4**. Scores for mean Object Masking performance averaged across ISIs were normally distributed and free of outliers. In ROSz, better Object Masking performance was associated with better social cognitive task performance. This was true for both low- and high-level tasks, and the correlations were of similar magnitude (*r*_EmoBio_= 0.45, *p* = 0.01; *r*_TASIT_ = 0.41, *p* = 0.02).

Regression analyses were conducted to test whether the relationship between visual processing and higher-level social cognition (i.e., TASIT part 3 performance) was mediated by lower-level social cognition (i.e., Emo Bio). The data were free of multivariate outliers. Object Masking performance was a significant predictor of the proposed mediator [Emo Bio, β = 0.45, *t* (29) = 2.70, *p* < 0.001] and the outcome variable [TASIT, β = 0.41, *t* (29) = 2.41, *p* = 0.02]. However, Emo Bio performance was not a significant predictor of TASIT performance [β = 0.28, *t* (29) = 1.58, *p* = 0.13]. Thus, these data do not provide significant support for mediation.^2^

## Discussion

In this study, we evaluated early visual processing, assessed with visual backward masking, and social cognitive task performance in people with recent-onset schizophrenia (ROSz) and healthy controls (HC). We hypothesized that visual backward masking performance and social cognitive task performance would be significantly impaired in ROSz compared to HC. In addition, we hypothesized that visual backward masking performance would be significantly correlated with social cognitive task performance in the ROSz group. Our first hypothesis was supported. Congruent with the findings of Favrod et al. (25) and Perez et al. (22), early visual processing was significantly impaired in the early phase of schizophrenia. The masking effect was exaggerated in the ROSz group. Compared to HC, the ROSz group showed greater interference from the mask, as indicated by lower performance accuracy across ISIs, and the patient group required longer ISIs to escape the masking effect. Similarly, we found support for our second hypothesis: social cognition was significantly impaired in ROSz. The ROSz group exhibited an impaired ability to identify emotional states from body movement cues (low-level social cognition), and impaired theory of mind (high-level social cognition).

An association between visual processing and social cognition has previously been demonstrated in chronic phase schizophrenia (7–14). This relationship is hypothesized to reflect a cascading effect of disruptions of early stages of information processing on downstream cognitive functions (7, 12, 44). Consistent with a cascade model, early visual processing was associated with performance on the social cognitive tasks in this ROSz sample. Thus, our third hypothesis was supported. Consistent with prior findings in chronic schizophrenia (7, 9–14), the strength of the association between visual processing and social cognition was moderate in magnitude. Moreover, the relationship was consistent across low- and high-level social cognition processes.

Although the data supported a cascading effect between visual processing and social cognition, a cascading effect *within* social cognition, i.e., with low-level social cognition performance predicting high-level social cognition, was not supported by the regression analyses. Our results are in contrast to theoretical accounts and empirical data for a hierarchical stream within social information processing (45, 46). However, a major limitation of the current study was the small sample size, which likely rendered the analyses underpowered to detect mediation.

Remediation of the perceptual and cognitive impairments associated with schizophrenia using a neuroplasticity-based, bottom-up training approach is a growing area of research, and the visual system is amenable to training. The studies conducted so far suggest that targeted visual training holds promise for remediating visual processing impairments in people with schizophrenia. Improved performance on trained visual tasks, including visual backward masking, motion perception, contrast sensitivity, visual search efficiency, visual acuity, and perceptual organization have been reported (47–51). Beyond improvements on trained tasks, data from the present study suggest that interventions directed at improving early visual processing might also yield effects on downstream social cognitive processes in those with a recent onset of schizophrenia. This is a question for future research.

## Conclusions

Early-stage visual processing, low-level, and high-level social cognition were all significantly impaired in ROSz. These data provide support for a cascade model of information processing between early-stage visual processing and social cognition in ROSz, but did not support significant cascading effects within social cognition.

## Data Availability Statement

The datasets generated for this study are available on request to the corresponding author.

## Ethics Statement

The studies involving human participants were reviewed and approved by UCLA Institutional Review Board. The patients/participants provided their written informed consent to participate in this study.

## Author Contributions

AM, MG, JW, and KN contributed conception and design of the study. AM managed data collection, organized the database, performed the statistical analysis, and wrote the first draft of the manuscript. JW, JL, ER, and MG wrote sections of the manuscript. JV and KS managed subject recruitment, and clinical and cognitive characterization of the sample. All authors contributed to the article and approved the submitted version.

## Funding

This study was supported by an individual fellowship award from the Canadian Institutes of Health Research (CIHR MFE120919, PI: AM) and by a NIMH Center Grant (P50 MH066286, PI: KN). AM is supported by a career development award from the NIMH (K23 MH108829).

## Conflict of Interest

AM has received compensation from MedAvante-Prophase, Inc. for clinical assessment services unrelated to this project, and she has received research support from Alkermes, Inc. ER was previously an employee of Data Cubed LLC. JV has received funding from Brain Plasticity, Inc., Genentech, Inc., and has served as a consultant to Boehringer-Ingelheim, GmbH, and Brain Plasticity, Inc. KS has served on the speakers bureau for Otsuka’s Abilify Maintena, a CME speaker for Janssen Inc. Canada and PeerVoice. He has served as a consultant to Janssen Scientific Affairs, LLC, Alkermes, Inc., Medincell, Inc., and Teva Pharmaceuticals, and has received research support from Alkermes, Inc. MG and KN are officers within MATRICS Assessment, Inc., the publisher of the MCCB, but do not receive any financial remuneration for their roles. MG has been a consultant to Biogen, Click Therapeutics, Lundbeck, and Roche, and he is on the scientific board of Cadent. KN has received research grants from Janssen Scientific Affairs, LLC, Posit Science, Inc., and Genentech, Inc., and has been a consultant to Astellas, Biogen, Genentech, Janssen, Medincell, Otsuka, Takeda, and Teva.

The remaining authors declare that the research was conducted in the absence of any commercial or financial relationships that could be construed as a potential conflict of interest.

## Footnotes

1 Note: Results from a follow-up repeated measures analyses in the ROSz sample that included CPZ equivalents as a covariate did not differ substantively from those reported in the manuscript (main effect of ISI, *p* < 0.001; main effect of CPZ, *p* = 0.11, ISI x CPZ, *p* = 0.68).

2 Note: Subsequent analyses that included CPZ equivalents did not substantively differ from the results reported above. Specifically, the association between OM and the two social cognition measures remained statistically significant after controlling for CPZ equivalents.

## References

[1] Jahshan C Wynn JK McCleery A Glahn DC Altshuler LL Green MF Cross-diagnostic comparison of visual processing in bipolar disorder and schizophrenia. J Psychiatr Res (2014) 51:42–8. 10.1016/j.jpsychires.2013.12.014 PMC4519235 24433849

[2] Butler PD Javitt DC Early-stage visual processing deficits in schizophrenia. Curr Opin Psychiatry (2005) 18:151–7. 10.1097/00001504-200503000-00008 PMC1994776 16639168

[3] Braff DL Saccuzzo DP Geyer MA Information processing dysfunctions in schizophrenia: studies of visual backward masking, sensorimotor gating, and habituation. In: Steinhauer SR, Gruzelier JH, Zubin J, editors, Handbook of schizophrenia, Vol. 5. Neuropsychology, psychophysiology, and information processing. Elsevier Science (1991). p. 303–334. 10.1017/S0033291700039167

[4] Cadenhead KS Serper Y Braff DL Transient versus sustained visual channels in the visual backward masking deficits of schizophrenia patients. Biol Psychiatry (1998) 43:132–8. 10.1016/S0006-3223(97)00316-8 9474445

[5] Rund BR Egeland J Sundet K Asbjørnsen A Hugdahl K Landrø NI Early visual information processing in schizophrenia compared to recurrent depression. Schizophr Res (2004) 68:111–8. 10.1016/S0920-9964(03)00193-2 15099595

[6] Green MF Nuechterlein KH Mintz J Backward Masking in Schizophrenia and Mania: I. Specifying a Mechanism. Arch Gen Psychiatry (1994) 51:939–44. 10.1001/archpsyc.1994.03950120011003 7979881

[7] Green MF Hellemann G Horan WP Lee J Wynn JK From perception to functional outcome in schizophrenia: modeling the role of ability and motivation. Arch Gen Psychiatry (2012) 69:1216–24. 10.1001/archgenpsychiatry.2012.652 PMC3976993 23026889

[8] Sergi MJ Green MF Social perception and early visual processing in schizophrenia. Schizophr Res (2003) 59:233–41. 10.1016/S0920-9964(01)00405-4 12414080

[9] Brittain P ffytche DH McKendrick A Surguladze S Visual processing, social cognition and functional outcome in schizophrenia. Psychiatry Res (2010) 178:270–5. 10.1016/j.psychres.2009.09.013 20494457

[10] Rassovsky Y Horan WP Lee J Sergi MJ Green MF Pathways between early visual processing and functional outcome in schizophrenia. Psychol Med (2011) 41:487–97. 10.1017/S0033291710001054 PMC5534526 20482936

[11] Matsumoto Y Takahashi H Murai T Takahashi H Visual processing and social cognition in schizophrenia: Relationships among eye movements, biological motion perception, and empathy. Neurosci Res (2015) 90:95–100. 10.1016/j.neures.2014.10.011 25449145

[12] Sergi MJ Rassovsky Y Nuechterlein KH Green MF Social perception as a mediator of the influence of early visual processing on functional status in schizophrenia. Am J Psychiatry (2006) 163:448–54. 10.1176/appi.ajp.163.3.448 16513866

[13] Norton D McBain R Holt DJ Ongur D Chen Y Association of Impaired Facial Affect Recognition with Basic Facial and Visual Processing Deficits in Schizophrenia. Biol Psychiatry (2009) 65:1094–8. 10.1016/j.biopsych.2009.01.026 19268917

[14] Kelemen O Erdélyi R Pataki I Benedek G Janka Z Kéri S Theory of mind and motion perception in schizophrenia. Neuropsychology (2005) 19:494–500. 10.1037/0894-4105.19.4.494 16060824

[15] Rassovsky Y Green MF Nuechterlein KH Breitmeyer BG Mintz J Visual processing in schizophrenia: Structural equation modeling of visual masking performance. Schizophr Res (2005) 78:251–60. 10.1016/j.schres.2005.05.011 15975768

[16] Savla GN Vella L Armstrong CC Penn DL Twamley EW Deficits in domains of social cognition in schizophrenia: a meta-analysis of the empirical evidence. Schizophr Bull (2013) 39:979–92. 10.1093/schbul/sbs080 PMC3756768 22949733

[17] Bora E Yucel M Pantelis C Theory of mind impairment in schizophrenia: meta-analysis. Schizophr Res (2009) 109:1–9. 10.1016/j.schres.2008.12.020 19195844

[18] Bora E Pantelis C Theory of mind impairments in first-episode psychosis, individuals at ultra-high risk for psychosis and in first-degree relatives of schizophrenia: systematic review and meta-analysis. Schizophr Res (2013) 144:31–6. 10.1016/j.schres.2012.12.013 23347949

[19] Keane BP Paterno D Kastner S Silverstein SM Visual integration dysfunction in schizophrenia arises by the first psychotic episode and worsens with illness duration. J Abnorm Psychol (2016) 125:43–549. 10.1037/abn0000157 PMC4850085 27030995

[20] Silverstein S Uhlhaas PJ Essex B Halpin S Schall U Carr V Perceptual organization in first episode schizophrenia and ultra-high-risk states. Schizophr Res (2006) 83:41–52. 10.1016/j.schres.2006.01.003 16497484

[21] Lencer R Keedy SK Reilly JL McDonough BE Harris MSH Sprenger A Altered transfer of visual motion information to parietal association cortex in untreated first-episode psychosis: Implications for pursuit eye tracking. Psychiatry Res - Neuroimaging (2011) 194:30–8. 10.1016/j.pscychresns.2011.06.011 PMC3185164 21873035

[22] Perez VB Shafer KM Cadenhead KS Visual information processing dysfunction across the developmental course of early psychosis. Psychol Med (2012) 42:2167–79. 10.1017/S0033291712000426 PMC4113431 22717191

[23] Lee J Nuechterlein KH Subotnik KL Sugar CA Ventura J Gretchen-Doorly D Stability of visual masking performance in recent-onset schizophrenia: An 18-month longitudinal study. Schizophr Res (2008) 103:266–74. 10.1016/j.schres.2008.03.005 PMC2859819 18450427

[24] Feigenson KA Keane BP Roché MW Silverstein SM Contour integration impairment in schizophrenia and first episode psychosis: State or trait? Schizophr Res (2014) 159:515–20. 10.1016/j.schres.2014.09.028 PMC4254521 25306205

[25] Favrod O Roinishvili M da Cruz JR Brand A Okruashvili M Gamkrelidze T Electrophysiological correlates of visual backward masking in patients with first episode psychosis. Psychiatry Res - Neuroimaging (2018) 282:64–72. 10.1016/j.pscychresns.2018.10.008 30415176

[26] Green MF Lee J Wynn JK Mathis KI Visual Masking in Schizophrenia: Overview and Theoretical Implications. Schizophr Bull (2011) 37:700–8. 10.1093/schbul/sbr051 PMC3122285 21606322

[27] Cuesta MJ García de Jalón E Campos MS Ibáñez B Sánchez-Torres AM Peralta V Duration of untreated negative and positive symptoms of psychosis and cognitive impairment in first episode psychosis. Schizophr Res (2012) 141:222–7. 10.1016/j.schres.2012.08.019 22989921

[28] American Psychiatric Association Diagnostic and statistical manual of mental disorders, 4th ed. (DSM-IV). Washington, DC: American Psychiatric Association (1994).

[29] Rothe PH Heres S Leucht S Dose equivalents for second generation long-acting injectable antipsychotics: The minimum effective dose method. Schizophr Res (2018) 193:23–8. 10.1016/j.schres.2017.07.033 28735640

[30] Andreasen NC Pressler M Nopoulos P Miller D Ho B-C Antipsychotic dose equivalents and dose-years: a standardized method for comparing exposure to different drugs. Biol Psychiatry (2010) 67:255–62. 10.1016/j.biopsych.2009.08.040 PMC3677042 19897178

[31] First MB Gibbon M Spitzer RL Benjamin LS Williams JB Structured clinical interview for DSM-IV® axis II personality disorders SCID-II. American Psychiatric Pub. (1997).

[32] Benjamin L Structured Clinical Interview for DSM-IV Axis II personality disorders (SCID II). New York NY Biom Res Dep (1994).

[33] Lukoff D Nuechterlein KH Ventura J Manual for the expanded brief psychiatric rating scale. Schizophr Bull (1986) 12:594–602. 10.1093/schbul/12.4.578

[34] Ventura J Green MF Shaner A Liberman RP Training and quality assurance with the Brief Psychiatric Rating Scale: “The drift busters”. Int J Methods Psychiatr Res (1993) 3:221–44.

[35] Nuechterlein KH Green MF MCCB: MATRICS Consensus Cognitive Battery. Matrics Assess Inc (2006).

[36] Reavis EA Lee J Wynn JK Narr KL Njau SN Engel SA Linking optic radiation volume to visual perception in schizophrenia and bipolar disorder. Schizophr Res (2017) 190:102–6. 10.1016/j.schres.2017.03.027 PMC5600632 28318839

[37] Kern RS Penn DL Lee J Horan WP Reise SP Ochsner KN Adapting social neuroscience measures for schizophrenia clinical trials, Part 2: trolling the depths of psychometric properties. Schizophr Bull (2013) 39:1201–10. 10.1093/schbul/sbt127 PMC3796089 24072805

[38] McDonald S Flanagan S Rollins J Kinch J A new clinical tool for assessing social perception after traumatic brain injury. J Head Trauma Rehabil (2003) 18:219–38. 10.1097/00001199-200305000-00001 12802165

[39] Heberlein AS Adolphs R Tranel D Damasio H Cortical regions for judgments of emotions and personality traits from point-light walkers. J Cognit Neurosci (2004) 16:1143–58. 10.1162/0898929041920423 15453970

[40] Cohen J Statistical power analysis for the behavioral sciences. Hillsdale, N.J.: L. Erlbaum Associates (1988). 10.1234/12345678.

[41] Tabachnick BG Fidell LS Using multivariate statistics. Pearson Boston, MA: Allyn & Bacon/Pearson Education (2007).

[42] Nuechterlein KH Miklowitz DJ Ventura J Gitlin MJ Stoddard M Lukoff D Classifying episodes in schizophrenia and bipolar disorder: Criteria for relapse and remission applied to recent-onset samples. Psychiatry Res (2006) 144:153–66. 10.1016/j.psychres.2004.04.018 17011635

[43] Butler PD Thompson JL Seitz AR Deveau J Silverstein SM Visual perceptual remediation for individuals with schizophrenia: Rationale, method, and three case studies. Psychiatr Rehabil J (2017) 40:43–52. 10.1037/prj0000212 27547852 PMC5322250

[44] McCleery A Ventura J Kern RS Subotnik KL Gretchen-Doorly D Green MF Cognitive functioning in first-episode schizophrenia: MATRICS Consensus Cognitive Battery (MCCB) Profile of Impairment. Schizophr Res (2014) 157:33–9. 10.1016/j.schres.2014.04.039 PMC4112962 24888526

[45] Javitt DC When Doors of Perception Close: Bottom-up Models of Disrupted Cognition in Schizophrenia. Annu Rev Clin Psychol (2009) 5:249–75. 10.1146/annurev.clinpsy.032408.153502 PMC4501390 19327031

[46] Ochsner KN The Social-Emotional Processing Stream: Five Core Constructs and Their Translational Potential for Schizophrenia and Beyond. Biol Psychiatry (2008) 64:48–61. 10.1016/j.biopsych.2008.04.024 18549876 PMC2453243

[47] Vaskinn A Andersson S Østefjells T Andreassen OA Sundet K Emotion perception, non-social cognition and symptoms as predictors of theory of mind in schizophrenia. Compr Psychiatry (2018) 85:1–7. 10.1016/j.comppsych.2018.05.002 29906670

[48] Norton DJ Hospital M Mcbain RK Ongur D Chen Y Perceptual training strongly improves visual motion perception in schizophrenia. Brain Cog (2011) 77(2):248–56. 10.1016/j.bandc.2011.08.003 PMC3195882 21872380

[49] Surti TS Wexler BE A pilot and feasibility study of computer-based training for visual processing deficits in schizophrenia. Schizophr Res (2012) 142:248–9. 10.1016/j.schres.2012.09.013 23043873

[50] Surti TS Corbera S Bell MD Wexler BE Successful computer-based visual training specifically predicts visual memory enhancement over verbal memory improvement in schizophrenia. Schizophr Res (2011) 132:131–4. 10.1016/j.schres.2011.06.031 PMC3195942 21795025

[51] Demmin DL Fradkin SI Silverstein SM Remediation of Visual Processing Impairments in Schizophrenia: Where We Are and Where We Need to Be. Curr Behav Neurosci Rep (2019) 6:13–20. 10.1007/s40473-019-00171-8

